# BDNF Impact on Biological Markers of Depression—Role of Physical Exercise and Training

**DOI:** 10.3390/ijerph18147553

**Published:** 2021-07-15

**Authors:** Eugenia Murawska-Ciałowicz, Mona Wiatr, Maria Ciałowicz, Gilmara Gomes de Assis, Wojciech Borowicz, Silvia Rocha-Rodrigues, Małgorzata Paprocka-Borowicz, Adilson Marques

**Affiliations:** 1 Physiology and Biochemistry Department, University School of Physical Education, 51-612 Wroclaw, Poland; 2 Department of Physiotherapy, Physiotherapy Faculty, Medical University in Wroclaw, 50-355 Wroclaw, Poland; mona.wiatr@gmail.com (M.W.); malgorzata.paprocka-borowicz@umed.wroc.pl (M.P.-B.); 3 Physiotherapy Faculty, University School of Physical Education, 51-612 Wroclaw, Poland; marysia.cialowicz@gmail.com; 4 Department of Molecular Biology, Gdansk University of Physical Education and Sport, 80-336 Gdansk, Poland; gilmara.gomesdeassis@awf.gda.pl; 5 Neurological Diseases Department, Medical University in Wroclaw, 51-618 Wroclaw, Poland; wojciech.borowicz@student.umed.wroc.pl; 6 Escola Superior de Desporto e Lazer, Instituto Politécnico de Viana do Castelo, Rua Escola Industrial Comercial de Nun’Álvares, 4900-347 Viana do Castelo, Portugal; silviadarocharodrigues@gmail.com; 7 Health Sciences and Human Development (CIDESD), Research Centre in Sports Sciences, Quinta de Prados, Edifício Ciências de Desporto, 5001-801 Vila Real, Portugal; 8 Tumor & Microenvironment Interactions Group, i3S, Rua Alfredo Allen 208, 4200-135 Porto, Portugal; 9 CIPER, Faculty of Human Kinetics, University of Lisboa, 1499-002 Cruz Quebrada, Portugal; amarques@fmh.ulisboa.pt

**Keywords:** depression, BDNF, physical activity, exercise training, myokines, mood, cognition, theories of depression

## Abstract

Depression is the most common and devastating psychiatric disorder in the world. Its symptoms, especially during the pandemic, are observed in all age groups. Exercise training (ET) is well known as a non-pharmacological strategy to alleviate clinical depression. The brain-derived neurotrophic factor (BDNF) is one of the biological factors whose expression and secretion are intensified in response to ET. BDNF is also secreted by contracted skeletal muscle that likely exerts para-, auto- and endocrine effects, supporting the crosstalk between skeletal muscle and other distant organs/tissues, such as the nervous system. This finding suggests that they communicate and work together to induce improvements on mood, cognition, and learning processes as BDNF is the main player in the neurogenesis, growth, and survival of neurons. Therefore, BDNF has been recognized as a therapeutic factor in clinical depression, especially in response to ET. The underlying mechanisms through which ET impacts depression are varied. The aim of this review was to provide information of the biological markers of depression such as monoamines, tryptophan, endocannabinoids, markers of inflammatory processes (oxidative stress and cytokines) stress and sex hormones and their relationship to BDNF. In addition, we reviewed the effects of ET on BNDF expression and how it impacts depression as well as the potential mechanisms mediating this process, providing a better understanding of underlying ET-related mechanisms in depression.

## 1. Introduction

Depression is currently the fourth most common health problem worldwide and it will be shortly more prevalent, due to the Covid-19 pandemic, according to the World Health Organization [1]. Today, over 350 million people worldwide suffer from depression, accounting for 4.4% of the population. Including masked depression, this proportion rises up to 10%, especially in the adult population. The disease affects mainly adults and the incidence depends on the age and region, being twice as often in women than men. The symptoms of depression, described as chronic sadness, lack of interest, and pleasure in activities, are commonly associated with other somatic diseases, meaning that approximately 10% of all adults experience the symptoms during a year [1,2,3,4,5]. Hu et al. [3] report that in 1200 participants from China taking part in a study between January and February of 2020, 54% of responders reported they suffered moderate to severe psychological impacts because of the Covid-19 pandemic, one-third of them suffered from moderate to severe anxiety symptoms, and 17% obvious depressive symptoms. Eating and sleep disorders, permanent fatigue, and lack of concentration are also reported [4]. Therefore, patients with depression require support from their relatives and therapists, and very often pharmacological intervention [6]. In recent decades, a great deal of knowledge has been accumulated about the neurological mechanisms involved in depression, including the influence of inflammatory factors, the role of monoamines, and the influence of vascular disorders and genetics on the physiopathology of depression, regardless of environmental factors, eventually evolving to malfunction and/or damage to specific neural networks [7,8,9,10,11]. The datasets of gene regulation in depression is created and a combined portrait was created for men and women only [12]. Impairment of adult neurogenesis, altered nervous processes and synaptic depression, and neuronal atrophy are just some of the neuroplasticity-related processes that are at risk in patients with depression during clinical and experimental studies [13,14,15]. 

The central mediator of neuronal plasticity, brain-derived neurotrophic factor (BDNF), has been extensively studied for its role in synaptic formation and maintenance, and for the ability of the central nervous system (CNS) to regenerate and adapt to possible damage [16,17]. Higher BDNF levels are associated with better cognitive and psychiatric states in both healthy subjects and depressed patients [18,19,20]. However, recent studies have noted significant reductions in BDNF levels in depressed patients [21]. It has also been documented that in elderly patients, reduction of BDNF in the blood has been recognized as a biological marker of memory deficits and cognitive processes [20,21,22,23,24,25]. A number of plausible physiological and neurological mechanisms/theories of depression have been described [2]. The oldest postulated and biological mechanism is monoamine deficiency (monoamine theory). Depression can also be explained by tryptophan deficiency and disruption of the kynurenine pathway [26], metabolic stress with oxidative stress [27,28], and hormonal, immunological disturbance [29]. Light deficiency and dysregulation of circadian rhythms by serotonin deficiency are also important factors [30,31]. In addition, depression in the elderly is caused by physiological failure of the body and deterioration of the functions of the brain and its neurotransmitters [29]. It is well documented that the aging brain retains a certain plasticity which can also be stimulated and corrected by physical activity; however, there is also evidence of the processes of atrophy of the brain structures with age. It has been shown that in people over 55 years of age without dementia the hippocampal atrophy ranges from 1–2%, prefrontal cortex, caudate nucleus and cerebellum 0.5–2%/a year, while stratum primary motor and sensorimotor regions remain rather unchanged [32].

The benefits of ET for the human body and mental health have been well documented. Regular ET has been described to be a preventive strategy against chronic disorders, such as type II diabetes mellitus, overweight/obesity, and atherosclerosis [33,34,35]. In fact, several benefits have been attributed to ET by improving cardiopulmonary function, stimulating skeletal muscle and bone tissue growth, reducing fat mass, and regulating hormone and whole-body lipid and glucose metabolism [35,36]. As a physiological regulator of BDNF production [18,37,38], ET has been revealed as an alternative strategy for the therapy and treatment of patients with disorders of nervous system, including depression [39,40]. Regular practice of ET facilitates everyday life, reduces everyday fatigue, positively influences mood and emotional processes such as anxiety and the feeling of being lost [41], and is currently recommended as an assessment strategy in the treatment and prevention of depression [42]. The effects of ET are widely recognized in brain function [43]; however, the underlying mechanisms by which ET exerts its effects as an antidepressant method are not fully understood. 

## 2. Brain-Derived Neurotrophic Factor

BDNF belongs to a family of growth factors that also includes the nerve growth factor, neurotrophin-3, and neurotrophin-4/5 [18]. BDNF is synthesized in the cell in the form of a precursor molecule called pro-BDNF, converted into mature BDNF form by post-translational cleavage, and then secreted into the extracellular space [44]. Both molecular forms, mature BDNF and pro-BDNF, are biologically active and act through specific receptors located in the cell nucleus and membrane [45]. BDNF binds to the tyrosine kinase B (TrkB) receptor and the p75 neurotrophins receptor (p75NTR). BDNF has a much greater affinity for the TrkB receptor than pro-BDNF, which, in turn, has a greater affinity for p75NTR [46,47]. While the activation of the TrkB receptor produces a protective and anti-apoptotic effect, the activation of p75NTR causes apoptotic and neurodegenerative processes [18,44,45]. The TrkB receptor is largely expressed in the neurons of the hippocampus [48].

According to Matthews et al. [49] and Rasmussen et al. [50], about 70–80% of circulating BDNF is produced in the brain at rest and in response to exercise training (ET). In the central nervous system, BDNF is mainly synthesized in the hippocampus, and also in the cerebral cortex, midbrain, thalamus (amygdaloidal body), hypothalamus, pons, or medulla oblongata [51,52]. Thus, BNDF is able to cross the blood-brain barrier in both directions and the variations of circulating BDNF concentration are likely come from both neurons and glial cells of the central nervous system [50].

In blood, BDNF is stored mainly in platelets (99%) [53,54], which explains its higher concentration in serum compared to plasma. Plasma BDNF levels decrease with age and body mass gain, as opposed to the constant concentration of BDNF in serum and platelets [55,56]. Other than by platelets, BDNF is also produced by T-cells, B-monocytes, and endothelium cells [57]. In plasma, there is only a small amount of circulating free BDNF [58].

In women, the BDNF content in platelets varies throughout the menstrual cycle. BDNF possibly is secreted by endometrium cells and its accumulation may be associated to the phase of menstrual cycle. In the follicular phase, the plasma BDNF accumulation is higher than luteal phase [56]. Moreover, menopause seems to have an impact on BDNF accumulation. Postmenopausal women showed lower levels of BDNF than premenopausal women [56]. Very low concentrations of BDNF have been observed in women who had not menstruated for at least 6 months, as compared with postmenopausal concentrations. These data suggest that there is an affinity between concentration of BDNF and sex hormones in women. It seems that the expression of the TrkB receptor, which demonstrates a large affinity for BDNF, is under a positive regulation influence of some sex steroids such as estrogen hormones [56,59,60]. 

In neurons, BDNF is present not only in the cytoplasm, but also near the dendritic spines, influencing their development [61]. BDNF stimulates the processes of neuroplasticity, which is manifested in neurogenesis, stimulation of the plasticity of serotoninergic, dopaminergic, cholinergic, or noradrenergic neurons, dendritogenesis, and synaptogenesis [62]. Additionally, BDNF facilitates the growth and survival of neurons and microglia cells. It also participates in the differentiation of cells, potentiation of signal transmission, induction, and the maintenance of long-term potentiation of synaptic enhancement [44,45,48,63]. Owing to these properties, BDNF improves cognition and takes part in emotional processes, spatial orientation, and learning, as well as body coordination [48,61,62,63,64,65,66].

The BDNF gene is a single nucleotide polymorphism that was found to substitute valine (Val) to methionine (Met) at position 66 in pre-pro-BDNF (Val66Met) [67]. The mutation can weaken the activity of the molecule and change the BDNF/pro-BDNF ratio. This single nucleotide polymorphism can influence the secretion of BDNF and is probably associated to neuropathology and cognitive deficiencies [67,68]. It has been stated that the mutation of Val66Met is correlated with depression occurrence. In patients with this mutation are characterized by a smaller hippocampus size and related memory impairments [68]. 

In patients with depression the DNA methylation of *BDNF* and negative relationship between serum BDNF level and miR-132/miR-182 levels are observed. An increase of miR-30e, miR-132, miR-185, and miR-212 in serum level was also observed in depressed patients [69].

## 3. Depression, Exercise, BDNF

**Figure 1:**
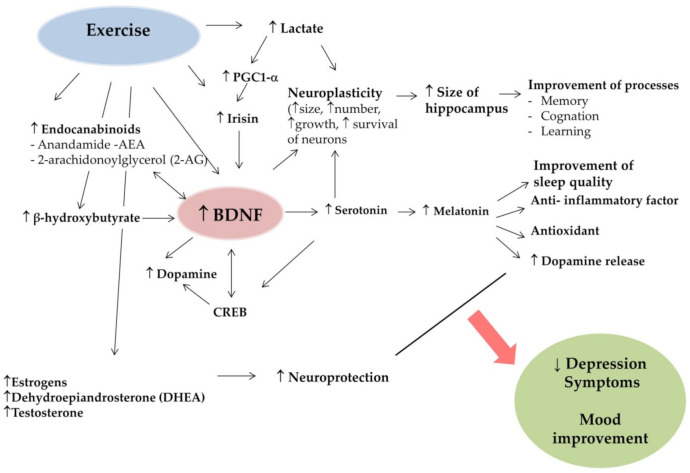
Potential mechanisms of ET impact on BDNF to decrease depression symptoms. (PGC1-α, peroxisome proliferator-activated receptor-gamma coactivator; BDNF, brain-derived neurotrophic factor; CREB, cAMP-response element binding protein).

Exercise training is recognized as a useful non-pharmacological strategy to improve the treatment of depression, and concomitantly decrease the somatic symptoms of this pathology [70]. Previous studies have shown that both endurance [22,30,71] and short-term high-intensity anaerobic exercise [72,73,74,75] can increase BDNF levels in healthy and depressed patients. According to Murawska – Ciałowicz et al. [76] BDNF levels after one bout of exercise are dependent on duration time, intensity, and type of test/exercise and the intensity of previous training. According to Rasmussen, BDNF is released from the brain during exercise [50] and is produced by skeletal muscle in response to contraction [49] and is responsible for muscle-brain crosstalk [77]. The influence of BDNF on biological mechanisms is described below and shown in Figure 1.

ET promotes favorable adaptations in the brain by improving learning, memory and cognitive processes in a depressed patient, as well as by an antidepressant effect [78]. However, the mechanisms behind this result are not clear and explained. It is thought that BDNF is a major factor since higher levels of this neurotrophin are seen after various ET modalities [79].

In depressed patients, ET increased blood BDNF [22,80,81,82]. In most of these studies, the increase of BDNF levels was also accompanied by an improvement in cognitive performance, processing speed, working memory, and sleep quality. The sparse BDNF studies in depressed patients participating in ET are shown in Table 1.

The most pronounced regulatory effect of ET on the brain is the oxygenation of the brain tissue because an increase in blood pressure had an impact on vascular endothelial activity and an increase in the diameter of the cerebral vessels [83]. Both endurance and resistance ET have been shown to increase the secretion of neurotransmitters (monoamines and noradrenaline), growth factors (vascular endothelial growth factor—VEGF, BDNF), and myokines by contracted skeletal muscle [84]. Skeletal muscle metabolites secreted into the bloodstream, a by-product of ET (lactate, β-hydroxybutyrate), also have a significant effect on brain tissue [43,85,86]. These metabolites are transported into cerebral circulation across the blood-brain barrier [43], and thus may contribute to several beneficial brain cellular processes [83].

Interestingly, the most spectacular effect is the neurogenesis of the hippocampus [48], which is the most essential component of neuronal plasticity, supporting the survival of neuronal cells and the regulation of synaptic plasticity [40,87]. ET-induced BDNF secretion has a positive regulatory effect on the neurogenesis-related mechanisms, stimulation of the activity of brain structures, and an increase in the size of the hippocampus [65,66,87].

## 4. Potential Mechanisms of Exercise Training Impact on BDNF in Healthy and Depressed Patients

ET constitutes a very strong stimulus to the brain and other organs and brings many beneficial health effects to the whole body on many levels of its organization, from the molecular level, through the cells, to the organs [93]. Data from numerous studies showed that ET potential protects in neurodegenerative diseases [42,94]. Research reveals that acute and chronic adaptations of exercise training have been related to stimulation of neurogenesis, which is essential for nervous system plasticity [43,95]. Exercise training stimulates new nerve connections and increases the number of synaptic vesicles. The higher secretion of neurotransmitters has also been reported [95]. Moreover, ET was able to induce substantial improvements in mating and memory processes, as well as in the emotional sphere [49,90,96].

In response to ET, constant monitoring of movement patterns is required from the brain, especially at the stage of learning new motor activities [41]. The beneficial influence of ET on brain health is manifested by the intensified blood supply to the brain, improvement in brain oxygenation, and activation of many regions of the brain structures. These facilitate better brain function, secretion of neurotransmitters that communicate at different regions of the brain, and secretion of different chemical substances with growth factor activity [43,83,97,98]. Besides the local effect of BDNF in the brain, some authors suggest that the brain is the major source of circulating BDNF at rest and during exercise [50]. In this sense, ET is an effective stimulus for BDNF synthesis as reported by several studies [22,71,89,92].

Skeletal muscle is widely recognized as an endocrine organ [77,97]. Contracted skeletal muscles produce and secrete various biologically active molecules, known as myokines, including BDNF [77,84,97,99,100]. They likely act in an endocrine-dependent manner to favor the communication between skeletal muscle and distant organs, creating a network that integrates several signals coming from various organs [77,84,97,101]. Therefore, the communication between skeletal muscle and the nervous system through BDNF may constitute one of the strongest factors that stimulates neurogenesis [99]. BDNF is responsible for organ communication and can act as auto-, para-, or hemocrine-like fashion. Thus, BDNF is an example of neurokine, myokine, and adipokine, with a wide range of action with other cytokines [77,99,102]. Of particular interest is the continuous flow of information between the skeletal muscles and the brain, known as crosstalk [97]. It is also speculated that BDNF and its receptor play a key role in the central regulation of the energy balance through the BDNF/TrkB axis in adipose tissue [103], suggesting an interesting role for the regulation of whole-body metabolism.

The impact of physical activity on BDNF secretion and its participation in cognitive processes’ improvement has been reported [104]. An increase of BDNF concentration has been observed after physical activities that are single but of varying intensity and time, single but intense, or systematic, aerobic, and anaerobic, and also those that last for several weeks or several months [104]. Moreover, the increase of BDNF concentration remains transitional and rapid as the levels return to resting values 10–60 min after the exercise training cessation [105,106].

Different molecular mechanisms have been proposed to explain how exercise/ET can impact on BDNF synthesis in brain and peripheral tissues. One of the suggested is an increase in Ca^2+^ concentration in neurons and activation of signaling pathways: mitogen-activated protein kinase/extracellular signal-regulated protein kinase (MAPK/ERK) and Ca^2 +^ /calmodulin-dependent protein kinase, which are responsible for CREB phosphorylation and CREB transcription activation, and, consequently, transcription of the BDNF gene [107,108]. ET resulted in an increase in the level of the activated transcription factor and CREB phosphorylated [109]. Together, these are signaling molecules that play critical roles in synaptic plasticity, including learning and memory. Furthermore, this constitutes an important pathway for the cytoskeleton protein synthesis, dendrites’ growth, and branching in hippocampal neurons as well as inhibition of apoptotic growth [110]. Both BDNF and TrkB are widely and strongly expressed in the human brain. Activation of TrkB upon attachment of BDNF activates many intracellular pathways, including the MAPK/ERK pathways [65,77,111,112]. ET can cause persistent increases in phosphorylated CREB and BDNF that continue throughout the exercise period [109]. According to Finkbeiner et al. [113] CREB is a major mediator of neurotrophins’ responses.

Wrann et al. [99] reported that BDNF secretion by the hippocampus was possible owing to the activation of the peroxisome proliferator-activated receptor-gamma coactivator (PGC-1α)/fibronectin type III domain-containing protein 5 (FNDC5) (irisin) pathway. PGC-1α increases as a result of AMP kinase (AMPk) activation by a decrease in adenosine triphosphate in the cell (↓ATP/AMP↑) [114,115]. Similar to PGC-1α, AMPK takes part in the intensification of oxidative processes, including initiation of mitochondrial biogenesis by stimulating uncoupling protein expression; this contributes to greater oxygen uptake and utilization by skeletal muscle cells [116]. BDNF stimulates AMPK expression acting as an autocrine or paracrine factor. Moreover, cathepsin B and irisin secreted by skeletal muscles can cross the blood-brain barrier and mediate BDNF expression in the hippocampus [117,118,119,120]. It is believed that the neuroprotective effect of BDNF results from the activation of the TrkB/MAPK/ERK1/2/IP3K/Akt pathway, which inhibits apoptosis, the neurotoxic effects of glutamate and nitric oxide, and the negative oxidative effects of stress, which damage neurons [65,105,107].

Sleiman et al. [86] suggested a mechanism according to which β-hydroxybutyrate, one of the metabolites classified as ketone bodies, forming during ET in conditions of oxygen deficiency, can activate BDNF promotors, promotor I in particular, and stimulate its secretion. The authors have observed that intraventricular injection of β-hydroxybutyrate resulted in increased BDNF protein expression in the mouse hippocampus, as well as the release of the TrkB receptor-dependent neurotransmitters.

Bergersen et al. [118] proposed very convincing and interesting mechanisms of a quite possible contribution of lactate in BDNF secretion. Lactate, as an ET metabolite, can activate several pathways leading to neuronal plasticity activation. It is also one of the important metabolites produced during exercise, and assists as a fuel for the brain [119]. In the brain, it is produced by astrocytes and was able to cross the blood-brain barrier from the periphery, and thus regulates many processes via specific monocarboxylate transporters (MCTs). These transporters are spread within the brain in neurons (MCT-2) or astrocytes (MCT-4) [120]. In the brain, lactate is transferred via MCTs to neurons, where it is converted to pyruvate for aerobic energy production in mitochondria. In neurons, lactate is essential for the maintenance of long-term synaptic enhancement, a phenomenon important in memory processes [121]. According to Descalzi et al. [121], memory formation, as well as long-term potentiation, requires energy, which, in line with the astrocyte-to-neuron lactate shuttle hypothesis, is formed from lactate transported from astrocytes to neurons and converted into pyruvate. These findings suggest that pyruvate and β-hydroxybutyrate can replace lactate if the transport of lactate is attenuated. Pyruvate and β-hydroxybutyrate can enter the Krebs cycle to produce energy. Newman and Verdin [122] maintain that β-hydroxybutyrate is not only an exercise metabolite but also a significant releasing molecule. Yang et al. [123] reported that lactate takes part in neuroplasticity through the expression of Arc, c-Fos, and Zif268 genes, as well as activation of the N-methyl-D-aspartate receptor and the Erk1/2 release cascade.

El Hayek et al. [124] report that lactate-dependent increase in BDNF concentration is associated with the activation of the sirtuin1 (SIRT1) deacetylase. SIRT1 increases the concentration of PGC-1α and the secretion of FNDC5, participating in the PGC-1α/FNDC5/BDNF pathway [52]. This mechanism explains the role of effort-generated lactate in improving spatial learning and memory processes. Systematically performed exercises can therefore reduce the tension of the nervous system, increase the concentration of substances that positively affect human emotions, and exert an antidepressant effect [125].

Considering all the findings explaining the effect of lactate on BDNF secretion and its effect on cognition, we obtain another important argument in favor of being active and participating in physical activity for health and recommending exercise as an important therapeutic factor in mood disorders.

## 5. Neurobiological Mechanisms of Depression and BDNF

### 5.1. Monoamines

**Figure 2:**
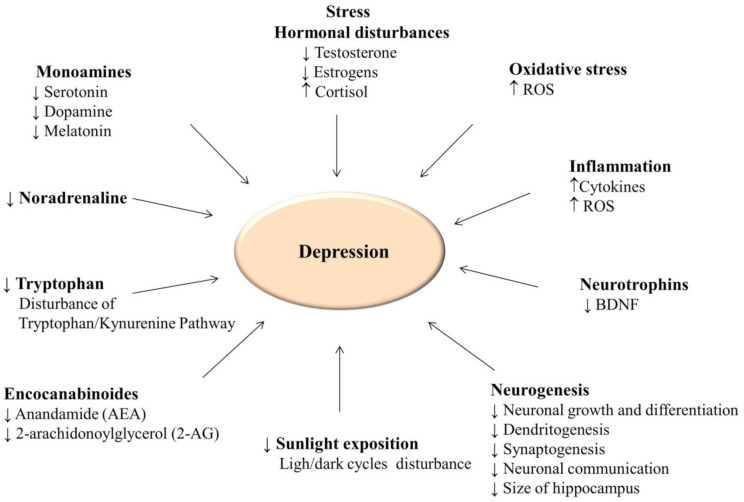
Biological markers of depression. (ROS, reactive oxygen species, BDNF, brain-derived neurotrophic factor).

Several biological mechanisms underlying depression are shown in Figure 2. One of the earliest explanations discussed was the monoamine theory, which relates to the deficiency or disturbance of catecholamine (dopamine and norepinephrine) and serotonin (5-hydroxytryptamine, 5-HT) secretion. In the light of this theory, depression is correlated with the depletion or imbalance in the secretion of these monoamines [126]. Depression has been treated with pharmacological agents such as selective serotonin and/or adrenaline reuptake inhibitors to improve neurotransmission impulses in key areas of the brain—the amygdala and hippocampus. However, at least 30% of depressed patients do not respond positively to antidepressants based on monoamine reabsorption inhibitors [7].

#### 5.1.1. Dopamine

This neurotransmitter, commonly known as the “pleasure hormone”, is the major catechol neurotransmitter that is produced and secreted by dopaminergic neurons in the human brain [127]. It is formed from the amino acid precursor of tyrosine that undergoes several downstream conversions before the release of dopamine. Dopamine binds to a series of receptors named dopamine receptors 1–5 (D1–D5) and participates in the regulation of processes related to locomotion, learning, feeling pleasure, and motivation [128]. Dopamine does not cross the blood-brain barrier; therefore, it must be synthesized in the central nervous system [129]. Increased dopamine concentration in the hypothalamus nucleus accumbens, which is a component of the “reward system” in the brain, seems to be a main biochemical mechanism of the “feeling of pleasure”, whereas disorders in dopamine production, secretion, and function are common causes of Parkinsonian diseases and schizophrenia [130].

It is thought that the dopamine system is responsible for drug addiction and the development of drug abstinence syndrome. Those conclusions come from studies in addiction to amphetamines and cocaine [129]. In depressed patients, a decreased activity of the nigrostriatal pathway, responsible for motor skills and a part of the extrapyramidal system, was found. Dopamine is degraded to biologically inactive homovanillic acid by enzymes from the group of monoamine oxidases (MAO-A and MAO-B), as well as catechol-O-methyltransferase [129]. BDNF has been shown to influence the release of dopamine in the mesolimbic dopamine system [131].

#### 5.1.2. Serotonin

Serotonin (5-hydroxytryptamine, 5-HT), commonly known as the “happiness hormone”, is an important neurotransmitter of the central nervous system. It is involved in mood, behavior, cognition, emotion, motor function, pain sensitivity regulation, and neuroendocrine regulations related to appetite, reproduction, circadian rhythms, and sleep since its metabolite is melatonin, the main regulator of sleep and rhythms [65,132]. Serotonin is a derivative of the exogenous amino acid tryptophan and exerts its biological effects through numerous receptors [132]. The antidepressant drug fluoxetine acts based on MAO inhibition in the synaptic cleft and the reuptake of serotonin [65]. Serotonin is the most essential factor involved in BDNF signaling, playing a very important role in the central nervous system, whereas its dysregulation is associated with different mental disorders [48,132].

Studies have shown that BDNF promotes the survival and morphological differentiation of 5-HT neurons and improves the functioning, sprouting, and growth of 5-HT neurons in various brain regions [133].

As indicated by Martinovich and Lu [65], serotonin stimulates the expression of the BDNF gene. BDNF has also been found to regulate the survival, development and function of serotonergic neurons, demonstrating the correlation between the two substances. Some authors have postulated synergy between BDNF and serotonin signaling systems and a feedback loop between BDNF and serotonin secretion [65,66,133]. In patients with depression, lower serum BDNF concentrations correlated with the severity of depression [29,66,134]. In addition, the cAMP response element binding protein (CREB) may also be involved in the loop between BDNF and serotonin. The role of CREB is important for long-term plasticity of neurons and increases the synthesis of L-dihydroxyphenylalanine (L-DOPA), a dopamine precursor [48]. CREB activity is serotonin dependent, while serotonin secretion is light dependent and is greater over long days [58,135]. Activated CREB stimulates BDNF expression, while BDNF promotes CREB activation through the TrkB receptor [135]. CREB is believed to be one of the molecular mechanisms of circadian rhythms that are dependent on light/dark rhythms [136,137,138,139].

### 5.2. Stress

Stress is one of the most important causes of depression. Mental stress (psychological stress), different than imposed by physical stress (e.g., ET), increases the risk of depression by several hormonal, biochemical, and immunological disturbances [140,141,142,143,144].

Studies suggest that both estrogen and testosterone play a significant role in protecting the nervous system and decreasing depression symptoms [142]. The release of glucocorticoids in response to stress is involved in the pathological mechanism of depression. Glucocorticoids exert their biological effects on gene expression through specific receptors [143]. Hippocampus and other cerebral areas express two adrenal steroid receptors – the mineralocorticoid receptor (MR, type 1) and the glucocorticoid receptor (GR, type 2) [144]. Stress stimulates the hypothalamic-pituitary-adrenal (HPA) axis and provokes the secretion by the hypothalamus of the corticotrophin-releasing hormone, which promotes the secretion of adrenocorticotropic hormone by the pituitary gland.

Adrenocorticotropic hormone induces the secretion of glucocorticoids by the adrenal glands and increases the concentration of these hormones in the blood and cerebrospinal fluid [145]. Higher concentrations of glucocorticoids, including cortisol, cause the reverse inhibition of corticotrophin-releasing factor secretion, which is called negative feedback.

Prolonged exposure to stress causes disturbances in the assessment of the HPA axis by increasing the concentration of glucocorticosteroids/cortisol in the blood and exerting a negative/destructive effect on the cells of the nervous system, which may stimulate the onset or intensification of depression [141]. Disturbances in the HPA axis and elevated cortisol levels have been observed in depressed patients [141,146,147]. As a result of the latter, the volume of the hippocampus decreases, which also causes negative feedback disturbance [7,141,148].

Many studies provide evidence of the relationship between stress, depression, impaired neurogenesis in the hippocampus, and the negative feedback between BDNF and cortisol [76]. HPA axis hyperactivity is considered one of the crucial biological factors of mood disorders, including depression [146,147,148,149]. Lower BDNF secretion in depressed patients may partly underlie the pathological mechanism of depression. BDNF secretion in the hippocampus is reduced in response to stress, while its concentration is increased after the intake of antidepressant drugs. Data from animals showed that BDNF injections improved their locomotor activity and temperature rhythm [150].

As mentioned earlier, the volume of the hippocampus is reduced in depressed patients [69]. The hippocampus is the part of the brain structure and limbic system that is responsible for learning and memory. It is especially accountable for memory processes – long-term and spatial memory, but also feeling emotions [141]. Its damage is possible in response to a prolonged stress. Such conclusions were drawn based on observing patients with Cushing syndrome, in which excess cortisol concentration is one of the symptoms [7].

It is believed that HPA axis disorders may lead to psychogenic depression. In men, this axis regulates the release of the steroid hormones testosterone and estradiol by the testes through a hormonal cascade involving the gonadotropin-releasing hormone, luteinizing hormone, and follicle-stimulating hormone. In patients with depression, the follicle-stimulating hormone and luteinizing hormone concentrations do not differ from those recorded in healthy men; estradiol concentration slightly increases, while testosterone concentration is significantly lower. In men, a decrease in testosterone concentration causes mood disorders, cognitive problems, or fatigue, because testosterone is a neuroactive hormone and its deficiency promotes the development of depression symptoms. Sex steroid hormones regulate neurogenesis in the hippocampus or the prefrontal cortex [151].

On the other hand, estrogen increased BDNF expression in different brain regions, e.g., olfactory bulb, hippocampus, cortex, or amygdala [142]. In ovariectomized female rats, estrogen treatment increased BDNF in the entorhinal cortex [142]. 17β-estradiol (17β-E2), a precursor of estrogen, has been shown to promote cell differentiation and survival in the culture of the hypothalamic, amygdala, and neocortical neurons. Moreover, 17β-E2 protects neurons against cell death caused by oxidative stress. This hormone appears to stimulate the same signaling pathways as BDNF [112]. The precursor of steroid hormone dehydroepiandosterone (DHEA) and its sulphate ester (DHEA-S) also have neuroprotective effects, interact with neurotrophins – BDNF, nerve growth factor, neurotrophin-3 – and, thus, stimulate the axon growth. DHEA produces its biological effects by binding to the tyrosine kinase A (TrkA) receptor and p75NTR in target cells. DHEA-S has been found to stimulate the sympathetic nervous system by both inhibiting gamma-aminobutyric acid and activating glutamate and N-methyl-D-aspartate receptor [152].

### 5.3. Stress, Neuroimmune Axis, and BDNF

In healthy individuals, hippocampus BDNF is very high, but stress significantly reduces its secretion. Antidepressants, in turn, improve BDNF secretion and intensify BDNF-dependent signaling pathways [39]. BDNF is one of the factors involved in the inflammatory process. Together with cytokines and chemokines, it cooperates in the regulation of the neuroimmune axis. According to Jin et al. [29], BDNF expression is affected by immune cell cytokines. The dysregulation of cytokine secretion in the brain intensifies inflammation and increases reactive oxygen species (ROS) production, which disrupts neuronal homeostasis and neurogenesis [2,153]. In fact, an administration of pro-inflammatory cytokines caused a significant reduction of BDNF gene expression [84].

Clinical observations showed that traumatic events, by activating the HPA axis, induced a stress response, activating many physiological mechanisms and intracellular pathways. Depression is usually a consequence of injuries, such as disturbances in the HPA axis, elevated cortisol concentrations, inflammatory processes, and oxidative stress [146].

A prolonged exposure to stress can cause neuronal degradation in various parts of the brain, especially in the limbic system. The interactions between the hippocampus and the amygdala and their mutual projections to areas of the prefrontal cortex are essential for effective emotional processes and the storage of important information in long-term memory [154].

### 5.4. Cytokines, Inflammation, and Oxidative Stress

The etiology of depression is still an open issue. The contribution of inflammation is obvious. Numerous studies reveal that depression is much more common among people suffering from chronic diseases with an ongoing inflammatory process. There is a close functional relationship between the brain and the endocrine and immune systems. The cells of the immune system and of various brain areas are equipped with hormone receptors through which hormones exert their biological effects. Moreover, the expression of receptors for several interleukins (IL) is observed in various parts of the brain, especially in the hippocampus and hypothalamus. In these areas, expression of IL-1, IL-2, IL-6, and tumor necrosis factor α receptors was demonstrated [2].

The cytokine theory of depression is supported by numerous studies in which an increase in the number of leukocytes, neutrophils, or macrophages. Pro-inflammatory substances secreted by these cells was observed, as well as changes in the subpopulations of T lymphocytes, an increase in the CD4/CD8+ lymphocyte ratio and concentration of acute-phase proteins of metalloproteinases [2].

Cytokines are thought to modulate neuroplasticity and alter the synthesis, reuptake, and metabolism of mood-regulating neurotransmitters. The participation of the inflammatory process in the development of mood disorders is also translated by cytokines into the modulation of synaptic plasticity and changes in the synthesis, reuptake, and metabolism of neurotransmitters involved in the regulation of mood [155].

Inflammatory cytokines have been proven to influence the synthesis and reuptake of serotonin, noradrenaline, and dopamine, whose disturbances are observed in depression 156]. In turn, the dose of IL-1 and IL-6 increases the secretion of corticoliberin and activates the HPA axis [2,155,156]. According to this theory, it is believed that the behavioral disturbances observed in depression, as well as disturbances in the secretion of neurotransmitters or stimulation of the HPA axis, are a consequence of the secretion of pro-inflammatory cytokines. An overproduction of cytokines or their dosing to animals causes behavioral reactions and neurochemical changes characteristic of the stress response. In addition, the overproduction of the corticotrophin-releasing hormone may secondarily increase cytokine secretion, disrupting the balance of pro-inflammatory and anti-inflammatory cytokines. These findings provide evidence that cytokines are involved in neurohormonal (neuroimmune-axis) and behavioral responses in depression [66,140].

Oxidative stress and ROS may contribute to the damage of neurons and adversely affect the synthesis of BDNF, because ROS cause oxidative modifications of proteins, lipids, and nucleic acids [28]. They destroy cell membranes and cell receptors and modify the activity of enzymes and genes. They disrupt the functions of cells and contribute to their death. Therefore, it is believed that oxidative stress also causes neuroprogression by interfering with neurotransmission, especially with regard to 5-HT signals [27]. In depressed patients, a decreased sensitivity of 5-HT receptors has been observed as a result of impaired neurogenesis of these neurons [28].

### 5.5. Neurotrophins

In depression, the variation of neurotrophins’ concentrations, especially BDNF, has been reported [22,23]. According to current knowledge, there are also other biological factors associated with the development of depression that may be the target of the therapeutic process [66]. Clinical studies show that depression may be associated with cell loss or atrophy, especially in the hippocampus and cortex [157]. The latest reports reveal relationships of mood disorders with insufficient secretion of neurokines, i.e., protein substances that act as growth factors stimulating neurons, e.g., for growth, differentiation, or production of dendritic spines [62,65]. Disorders of their secretion constitute the basis of a theory called the neurokine theory or neurotrophic theory, and the deficiency of neurokines can be the reason for neuronal decline. The most researched and most important factor in this theory is BDNF. It is essential for the growth, development, and survival of neurons. Brain imaging studies of depressed patients show that, in addition to changes in the volume of the limbic system area, the cell bodies of pyramidal neurons and glial cells are lost or reduced [158].

It is believed that stress, especially chronic stress, through cortisol secretion due to disruption of the HPA axis, can cause neuronal loss in the hippocampus and impaired/reduced production of neurons in the dentate gyrus, the main structure of the hippocampus responsible for neurogenesis [7]. More details on how antidepressants can act and influence emotional state and mood through BDNF and in light of the theory of neurogenesis and synaptic plasticity can be found in Harmer et al. [159].

### 5.6. Anandamide and 2-Arachidonoylglycerol

The human body produces molecules/chemicals with an effect similar to that of the main psychoactive substance of marijuana, tetrahydrocannabinol, exerting its biological influence through two main cannabinoid receptors: CB1 and CB2. These substances are called endocannabinoids [160]. The CB1 receptor is located mainly in the central nervous system in various structures: in the cortex, hippocampus, basal ganglia, amygdala, hypothalamus, cerebellum, and limbic system, regulating emotions, satisfaction, feelings of pleasure, and fear [161]. This system is important in the process of remembering and motivating. A large accumulation of CB1 was also found in the layer of pyramidal cells of the hippocampus responsible for the processes of learning and memory, and in the nucleus accumbens, which is part of the human reward system, the main system of behavior motivation, at the same time, being the basis of such phenomena as addiction. In turn, the CB2 receptor occurs mainly peripherally, also in the peripheral nervous system, but is mainly expressed on immune cells and the spleen [160,162].

There are two endocannabinoids produced in the greatest amount: N-arachidonoylethanolamine (AEA) (anandamide) and 2-arachidonoylglycerol (2-AG). Anandamide is found in small amounts in chocolate, producing similar effects to marijuana tetrahydrocannabinol [161,162]. Data from different studies showed that exercise activates the endocannabinoid system [163,164,165].

The word *Ananda* comes from Sanskrit and means joy, delight, and bliss [163]. In a study by Sparling et al. [163] that involved runners and cyclists, after a 50 min exercise with an intensity of 70–80% HRmax, a significant increase in the concentration of anandamide was noted in both groups. Raichlen et al. [165] received that the secretion of AEA is dependent on intensity. According to their study the most effective response of AEA to 30 min of exercise also occurs at an intensity of about 70–80% of AAMHR (age-adjusted maximum heart rate), accounted according to the Tanaka formula. Both teams believe that anandamide may be responsible for the analgesia during exercise, for alleviating pain, tension, and anxiety, and for well-being. Furthermore, it is anandamide rather than endorphins that are considered as one of possible mechanisms responsible for the phenomenon known in sports as “runner’s high”, i.e., a state of contentment, euphoria, and increased resistance to pain and fatigue; the molecular mass of endorphins is too high for them to freely cross the blood-brain barrier [162,164,165]. Anandamide and 2-AG are produced in postsynaptic neurons. AEA is formed of membrane phospholipid. The 2-AG precursor is diacylglycerol. As a result of its metabolism, under the influence of various enzymes, other bioactive compounds may be formed. Both endocannabinoids are found in the peripheral blood in equal concentrations, and the brain concentration of 2-AG is about 170 times higher [166]. Both AEA and 2-AG are synthesized “on-demand” from membrane phospholipids and released immediately without vesicle storage [167].

2-AG is a very important signaling mediator responsible for brain homeostasis and anti-inflammatory and neuroprotective effects [168]. Some of the neuroprotective effect of BDNF is mediated by 2-AG [169]. Heyman et al. [72] reported that anandamide might be one of the key elements that contribute to increase BDNF concentration during ET and delaying its return to the pre-exercise level, both immediately after exercise and during recovery, suggesting that anandamide produced during ET may be one of the antidepressant mechanisms.

### 5.7. Tryptophan Pathway/Kynurenine

The levels of serotonin and melatonin in the brain are closely related to the amount of tryptophan in the body. Serotonin is produced from tryptophan by hydroxylation and decarboxylation and then converted to melatonin [170].

Tryptophan is an essential amino acid. Its content in the body depends on the diet and lifestyle, especially stress exposure. Its main source is a diet rich in protein. Inducing a long-term state of tryptophan deficiency in the body may lead to mood disorders [99]. In the digestive tract it is absorbed and converted into serotonin; 95% of serotonin is thought to be composed of tryptophan, although only 1–2% of tryptophan is used to synthesize serotonin [170]. The second source of serotonin synthesis are neurons of the nerve plexuses, from which about 5% of serotonin is derived [26].

Increased activity of the HPA axis and the inflammatory process caused by cytokine synthesis disturb the metabolism of tryptophan and significantly reduce its concentration in the blood [2]. It has been proven that inflammatory markers—IL-1, IL-2, interferon—increase the activity of the enzyme indoleamine 2,3-dioxygenase (IDO) and increase the metabolism of tryptophan towards the formation of kynurenine and several of its metabolites, commonly known as kynurenines, at the cost of synthesis serotonin and melatonin [2]. The enzyme is found in the cells of various tissues, including neurons and astrocytes, as well as in the microglia. A significant correlation was found between IDO activity, inflammatory markers and the severity of depression [171]. It has also been shown that some metabolites of tryptophan, the kynurenine pathway, the concentration of which is increased by IDO activation, have an adverse effect on behavioral processes, including Kynurenine causes anxiety and depressive symptoms, and its metabolites exert neurotoxic and neurodegenerative effects [26,147,151].

## 6. Light, Sleep, and BDNF

According to studies, the development of depression is influenced by the lack or deficiency of daylight or insufficient exposure to the sun [172]. These factors can result in decreased activity of the serotonergic system and disturbances of circadian rhythms and sleep, which are often observed in people with depression [172,173]. Light therapy has been shown to have a similar effect on neurotransmitters as antidepressants or serotonergic stimulants and is widely used in clinical settings for the treatment of depression [174]. It seems that sleep disturbances associated with its elongation or shortening may be an important factor contributing to the development of depression. Interesting research on the circadian rhythm of BDNF secretion was presented by Begliuomini et al. [56]. In this study, they reported that BDNF levels were higher in the morning (8:00) and much lower at noon (lower than at 8:00), and lowest at midnight. Moreover, Molendijk et al. observed seasonal variability in BDNF secretion [30] and found a strong relationship between BDNF concentration and the amount of sunlight. The lowest BDNF concentrations were recorded between January and March. After this period of the year, BDNF levels continued to rise until August and then declined systematically. It should be noted that the levels observed in the fall remained slightly higher than in the January-May period.

## Figures and Tables

**Table 1:** Effects of exercise training on BDNF levels in depressed patients.

Reference	Subjects	ET intervention	Main Findings
Kurdi et al., 2019 [80]	*n* = 70 elderly women; 35 with depression vs. non-depressed women Age: ≥50 y	Aerobic training: 15min/day on treadmill; speed at 6 km/h Duration: 28 days	35.3% ↑ BDNF from initial value in depressed and 16.3% from initial value in non- depressed women
Gourgouvelis et al., 2018 [81]	*n* = 16 MDD patients and physical inactive; Age: 39.31 ± 7.02 y	Combined aerobic and strenght trainings: 20 min; 3x/week (1 session of aerobic and 2 sessions of strenght) Aerobic training: 60 min at 60 and 80% of their age-predicted HRmax Strenght training: 2/3 supersets (without rest) with 8–12 rpts; exercises for main muscle groups at 95% of the 10 RM Duration: 8 weeks	↑ BDNF levels ↑ 31% in VO_2_max during exercise ↓ depression symptoms ↑ sleep quality and cognitive function
Vedovelli et al., 2017 [82]	*n* = 31 women independent and non-demented subjects;Age: 80 to 97 y *n* = 22 subjects were submitted to an ET intervention	Combined aerobic and strenght trainings: 3x/week, 3 sets of 10 rpts and 30 s interval during 30 min. Resistance bands exercises. Intensity: 50% of 1RM initially; 75% of 1RM in 3^rd^ month At the end of session: 30 min walk with 75–85% HRmax Duration: 3 months	↑ BDNF ↓ depression symptoms ↑ cognitive performance ↑ muscle strenght of lower limbs and aerobic condtion
Kallies et al., 2019 [88]	*n* = 30 MDD outpatients (*n* = 17 women); *n* = 7 patients with a single depressive episode and *n* = 23 patients with current depressive disorder Age: 39.2 ± 11.4 y	One bout of aerobic exercise: Graded exercise test on a cycle ergometer starting at 25w with progression of 25w every 2 min, until exhaustion	↑ BDNF Larger BDNF increase in women with smaller number of platelets.
Laske et al., 2010 [22]	*n* = 35 elderly women Age: 61.1 ± 7.2 y with depressive episode of recurrent unipolar depression	One bout of aerobic exercise: Incremental exercise test on treadmill (initial walking speed 3km/h). Speed and inclination increased simultaneously every 3 min.	↑ BDNF (immediately after exercise cessation) At 30 min of recovery, BDNF lower in comparison to baseline levels and immediately after exercise cessation
Meyer et al., 2016 [89]	*n* = 24 women with depression Age: 38.6 ± 14.0 y	One bout of aerobic exercise: 30 min stationary bicycle with intensity: light (RPE = 11); moderate (RPE = 13); hard (RPE = 15) Blood taken before and within 10 min after completion of each session	↑ BDNF Main effect of measurement, but not an effect of intensity Acute improvement in depressed mood, but not intensity depended
Szuhany and Otto, 2020 [90]	*n* = 29 sedentary adults with MDD or PDD with a current major depressive episode Age: 18–65 y	Stretching exercise: 9 sessions of stretching behavioral activityDuration: 12 weeks Balke protocol consisting of 2-min stage with speed and grade increasing over time. BDNF collection occurred immediately prior to test completion, immediately following test completion and at 4^th^, 8^th^ and 16^th^ week	↑ BDNF at 4^th,^ 8^th^ and 16^th^ week
Dopp et al., 2020 [91]	*n* = 13 adolescents with depression and physically inactive	Aerobic exercise: 3 supervised aerobic sessions in the 1^st^ week, 2 supervised aerobic sessions in the 2^nd^ week, and 1 supervised aerobic session in the 3^rd^ week. Blood take pre- and post-intervention. Balke Fitness test at week 1 and 12 Duration: 12 weeks	After 12 weeks: ↑ BDNF ↓ depression symptoms ↓ CDRS-R score
Schuch et al., 2014 [92]	*n* = 26 depressed inpatients, RCT Age: 42.81 ± 12.4	Aerobic exercise: 3x/week, targeted dose of 16.5 kcal/kg/week of aerobic exercise, Single-stage submaximal treadmill walking test according Ebbeling	↑ BDNF after 2 weeks. = at discharge in comparison to 2 weeks.
Pereira et al., 2013 [71]	*n*= 451 community-dwelling older women, RCT Age: 65–89 y	Aerobic, strenght training and combinaed aerobic and strenght trainings: Strenght training: 3x/week, six exercises for major muscles at 50–75% RM; Aerobic training: 5-min warm-up followed by 40 min of aerobic exercises at 65% and 80% age-predicted maximum heart rate, 3x/week Duration: 10 weeks	Strenght training: ↑ BDNF Both aerobic and strenght trainings: ↓GDS score

BDNF, brain-derived neurotrophic factor; MDD, major depressive disorder; VO_2max_, maximal oxygen consumption; HRmax, maximal heart rate; RM, repetition maximum; PDD, persistent depressive disorders; CDRS-R score, children’s depression rating scale revised; RCT, randomized controlled trial; GDS, geriatric depression scale.

## Data Availability

Not applicable.
